# Field oriented control dataset of a 3-phase permanent magnet synchronous motor

**DOI:** 10.1016/j.dib.2023.109002

**Published:** 2023-02-24

**Authors:** Juan Camilo Nustes, Danilo Pietro Pau, Giambattista Gruosso

**Affiliations:** a STMicroelectronics, via C. Olivetti 2, Agrate Brianza, I-20864, Italy; b Department of Electronics, Information and Bioengineering, Politecnico di Milano, Milano, Piazza Leonardo da Vinci, 32-20133, Italy

**Keywords:** Motor control, Simulink, ID control, Neural networks

## Abstract

This paper presents a dataset of a 3-phase Permanent Magnet Synchronous Motor (PMSM) controlled by a Field Oriented Control (FOC) scheme. The data set was generated from a simulated FOC motor control environment developed in Simulink; the model is available in the public GitHub repository^1^. The dataset includes the motor response to various input signal shapes that are fed to the control scheme to verify the control capabilities when the motor is subjected to real life scenarios and corner conditions.

Motor control is one of the most widespread fields in control engineering as it is widely used in machine tools and robots, the FOC scheme is one of the most used control approaches thanks to its performance in speed and torque control, with the drawback of having to handcraft the Proportional-Integrative-Derivative (PID) regulators using Look Up Tables (LUT).

The test conditions are designed by setting a motor desired speed. Different input speed variations shapes are proposed as well as extreme scenarios where the linear behaviour of the PID regulator is challenged by applying fast and high magnitude speed variations so that the PID controller is not able to correctly follow the reference. The measured data includes both the outer and inner-loop signals of the FOC, which opens the possibility to develop non-linear control approaches such as Machine Learning (ML) and Neural Networks (NN) with different topologies to replace the linear controllers in the FOC scheme.

**Specifications Table**

**Table:** 

Subject	Control and Systems Engineering
Specific subject area	Motor Control
Type of data	Dataset files: *.mat files Motor response images: *.jpg files
How data were acquired	Developed Simulink environment considering the FOC scheme and a PMSM motor model. Different signals were applied as an input to the model and the internal signals were measured and stored into files by using matlab scripts.
Data format	Raw
Description of data collection	Data generated in a mathematical model of the 3-phase motor inside the Simulink environment. The simulated motor is a 7 poles PMSM, with a maximum speed of 15000rpm (Full parameters table in Section 2.1). Different input signals of the desired speed were considered.
Data source location	Data was generated at ST Microelectronics, Agrate Brianza, Italy
Data accessibility	Repository name: PMSM_FOC datastet Direct URL to data: https://data.mendeley.com/datasets/6tjkgtfnky/2

## Value of the Data

This dataset simulates the behaviour of a 3-phase PMSM motor controlled using the FOC scheme [1], the signal values of the rotating DQ frame voltage and current are gathered both for the reference and measured signal. The dataset also includes the signal value of the reference and measured speed which is a valuable tool for the development and validation of ML algorithms for motor control without the need of having a physical motor to produce data.

• It contains data generated for both real life scenarios and corner conditions to stress to the maximum the linear controllers (PID) deployed in the FOC scheme.
• Practitioners working on motor control applying a ML or NN approach can benefit greatly from this dataset, as it removes the need to dump measurements directly from a physical motor.
• The dataset can be used, for example, in ML tasks such as training, testing, and validating a NN. The dataset can be used to train and test various architectures of ML and NN based regressors.
• ML approaches can be adopted for defining the actual operating conditions of the controller [step-generated], and then test the performance when applying an extreme condition scenario [random-generated] [2], [3].

## 1 Objective

This dataset was created to mimic the response of a PMSM when subjected to a wide set of input speed shapes as well as test conditions specifically designed to highlight how the nonlinearities affect the PID controller present in the FOC scheme, in this way, a physical motor to gather data is no longer needed. The inclusion of non-linear boundary brings the possibility to design a non-linear control approach such as ML or NN based control, capable of matching and, if possible, outperforming the behavior of a PID regulator in certain situations [4].

## 2 Data Description

The dataset consists of .mat data arrays, along with .jpg images for each test case in order to easily preview the speed control outcomes. Each test case corresponds to a desired speed input scenario fed to the motor control scheme. The main folder Motor_Control_Dataset is divided in four branches, each one corresponding to the type of signal that the system has as input.

The first branch 1_Step_Input contains data gathered when a step input is applied to the system, the second branch presents data obtained through ramp inputs (2_Ramp_Input) with different slopes. The content of 3_Random_Signal and 4_Generated_Signal is a set of test cases based on a random and a graphically designed signal respectively.

The directory tree is organized as follows:

- Motor_Control_Dataset
  ○ 1_Step_Input
  ○ 2_Ramp_Input
  ○ 3_Random_Signal
  ○ 4_Generated_Signal

### 2.1 Step Input Branch

The subfolder 1_Step_Input contains five test cases obtained by setting a step input with the value of the desired speed of the motor. Each test folder contains the .mat data array as well as a .jpg image comparing the reference and measured speed. The data is normalized considering that the maximum motor speed is 15000rpm. The directory tree is shown below:

- Motor_Control_Dataset
  ○ 1_Step_Input
    ■ 25_step
      • 25_step.jpg
      • 25_step.mat
    ■ 40_step
    ■ 60_step
    ■ 80_step
    ■ 100_step
  ○ 2_Ramp_Input
  ○ 3_Random_Signal
  ○ 4_Generated_Signal

Each test case is named as it follows:

<max. speed percentage>_<input type>.extension

**Fig 1:**
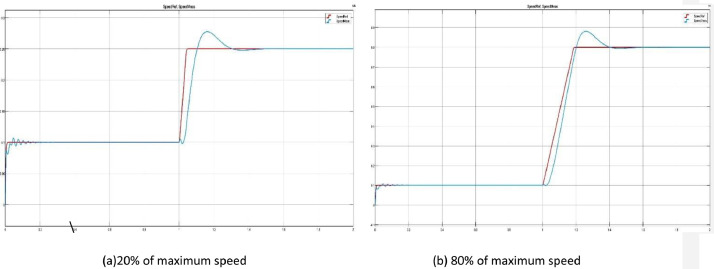
Motor speed measured (blue) vs reference (red) with a step input.

Fig. 1 shows examples of the .jpg files contained in two test folders, which are useful as a preview of the data array by showing the comparison between the reference and measured speed. Fig. 1a shows the step response for a target speed of 20% (3000 rpm), while Fig. 1b presents the response to a step of an 80% (12000 rpm) speed reference.

The description of every dataset parameter is reported in Table 2 and is valid for all dataset branches. An example of the test case 25_step.mat is shown in Table 2. The columns correspond to the parameters described in table 1.

**Table 1:** Description of the dataset parameters, valid for all four dataset branches, with reference to section 2.2.

Parameter	Abbreviation	Description	Measure unit
Speed Reference	SpeedRef	Desired speed of the motor, set by the user	%
Measured Speed	SpeedMeas	Actual speed of the motor product of the FOC	%
D current reference	Id_ref	Reference value of current for torque control, set to zero	A
Q current reference	Iq_ref	Reference value of current for torque control, obtained through the speed control regulator (outer loop)	A
Measured D current	Id_meas	Feedback current value obtained from Park transform	A
Measured Q current	Iq_meas	Feedback current value obtained from Park transform	A
Measured D voltage	Vd	Output voltage value from the PID torque regulator	V
Measured Q voltage	Vq	Output voltage value from the PID torque regulator	V

### 2.2 Ramp Input Branch

The directory structure is very similar to the step input branch and is reported below. The difference is the type of signal that will be input to the system, in this case is a ramp that will increase the motor speed from the open loop value (10% of max speed) with a constant slope. The test cases are defined by changing the slope value, which is expressed as a percentage of the maximum motor speed (15000 rpm).

- Motor_Control_Dataset
  ○ 1_Step_Input
  ○ 2_Ramp_Input
    ■ 10_ramp
      • 10_ramp.jpg
      • 10_ramp.mat
    ■ 20_ramp
    ■ 40_ramp
    ■ 80_ramp
    ■ 100_ramp
  ○ 3_Random_Signal
  ○ 4_Generated_Signal

**Fig 2:**
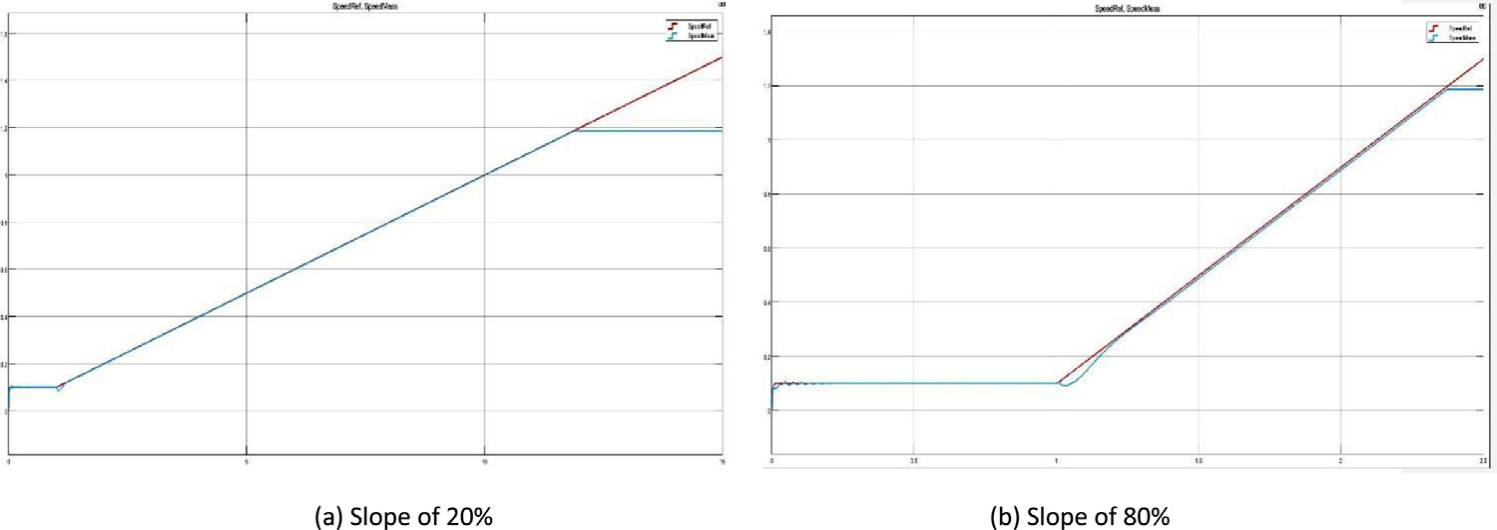
Motor speed measured (blue) vs reference (red) with a ramp input.

The ramp data set contains five different cases where the slope is varying from a 10% to a 100% slope value, changing the time needed to reach the maximum speed value that the motor can provide. Fig. 2a shows the ramp response for a slope of 20% (3000 rpm/s), while Fig. 2b presents the response to a ramp with a slope of 80% (12000 rpm/s).

### 2.3 Random Signal Input Branch

The third directory follows the same format as the previous two and it is shown below. In this case, the test cases are defined by applying a random signal generated around a random mean value. The motor runs for 10 seconds in each test considering the first second as the open loop start. The four test cases are named by considering the mean speed value around which the signal was generated (In this case: 20%, 40%, 70% and 100%). Multiple random test cases are considered to verify how the system reacts to fast speed changes, and if this behaviour varies when the mean value of the speed changes, recalling that the speed is normalized considering the maximum motor speed of 15000 rpm.

- Motor_Control_Dataset
  ○ 1_Step_Input
  ○ 2_Ramp_Input
  ○ 3_Random_Signal
    ■ 20_random
      • 20_random.jpg
      • 20_random.mat
    ■ 40_random
    ■ 70_random
    ■ 100_random
  ○ 4_Generated_Signal

**Fig 3:**
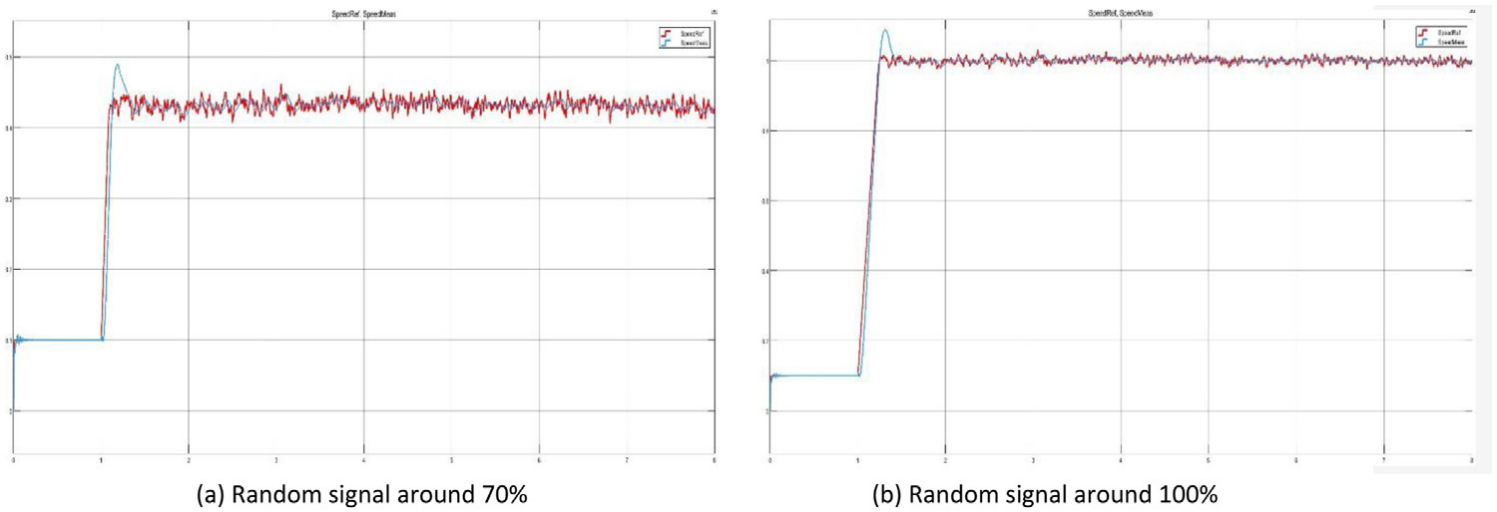
Motor speed measured (blue) vs reference (red) with a random signal generator.

In Fig. 3 are presented two examples of the .jpg files contained in the test folders, showing the behaviour of random signal generated around a mean value of a 70% (Fig. 3a), and Fig. 3b is generated around a 100% mean value (15000 rpm/s).

### 2.4 Graphically Designed Signal Input Branch

The fourth and last directory keeps the same format as the first three directories mentioned above. The difference with this branch is that the input signal is a graphically designed signal generated through the “signal builder” block in Simulink, where the user can design the shape of a signal by “drawing” the waveform. This input shape gives the possibility to the user to test the control performance of the system when subjected to a desired input, while also opening the possibility to test specific conditions to highlight the controller flaws to nonlinearities, which are present when the controller is subjected to high magnitude speed variations, or very fast transitions. The directory tree is shown below:

- Motor_Control_Dataset
  ○ 1_Step_Input
  ○ 2_Ramp_Input
  ○ 3_Random_Signal
  ○ 4_Generated_Signal
    ■ 1_generated
      • 1_generated.jpg
      • 1_generated.mat
    ■ 2_generated
    ■ 3_generated
    ■ 4_generated
    ■ 5_generated
    ■ 6_generated
    ■ 7_generated

**Fig 4:**
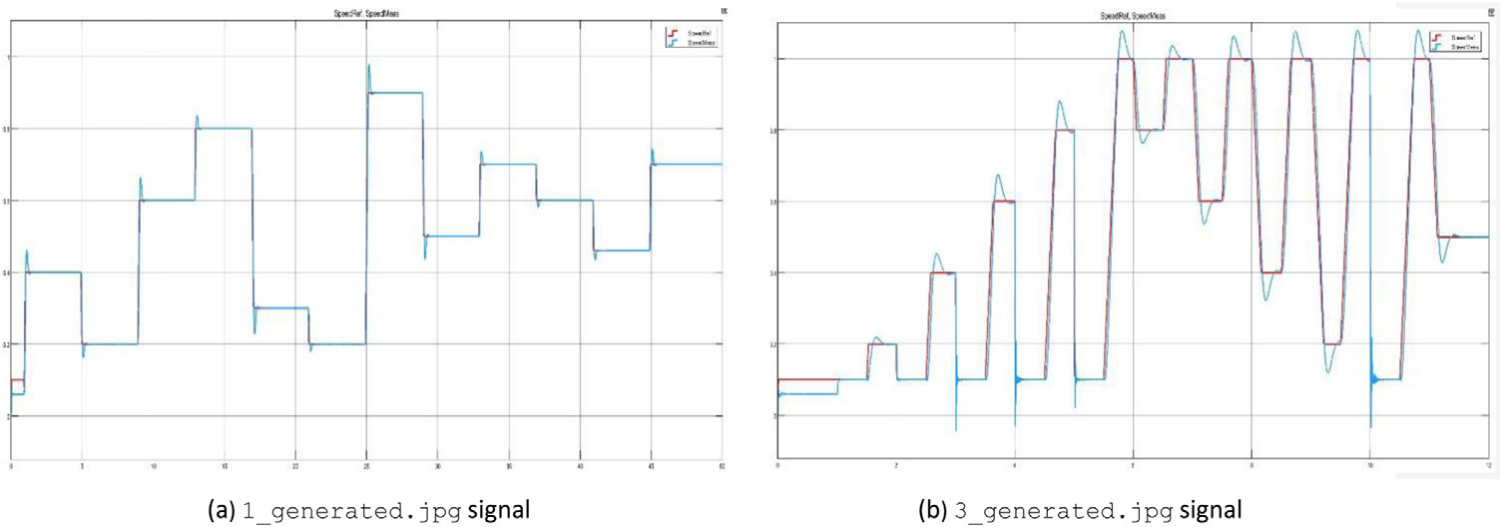
Motor speed measured (blue) vs reference (red) with a manually generated signal.

Fig. 4 shows examples of the .jpg files contained in two generated signal test folders, which are useful as a preview of the data array by showing the comparison between the reference and measured speed.

Fig. 4a shows the response for the test case 1_generated, while Fig. 4b presents the response to the test case 3_generated. Both signals were built to show the overshoot caused by high magnitude speed variations.

## 3 Experimental Design, Materials and Methods

The dataset is based on a simulated environment of a FOC scheme implemented in Simulink to control the speed of a 7 pole Permanent Magnet Synchronous Machine with a maximum speed of 15000rpm when no torque is applied. To gather data from the deigned environment, a test set of inputs was designed to verify how the controller reacts to the target speed. The Simulink model is available in [5].

### 3.1 Motor Model

**Fig. 5:**
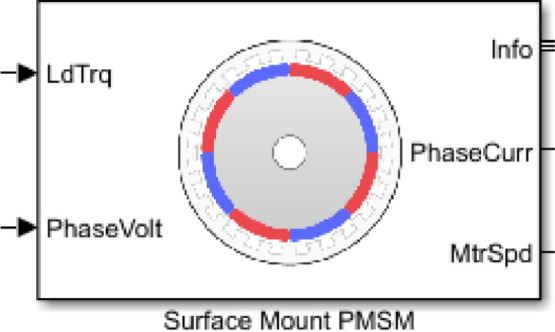
Surface mounted PMSM block.

For the model of the motor, the “Surface mounted PMSM” block (Fig. 5) was used, which is part of the Motor Control Block set provided by Matlab, it internally considers all the machine dynamics and the final transfer function. Then, this block was tuned with the parameters of the motor (Table 3) obtained through the Motor Profiler software available at^2^.

### 3.2 FOC Scheme

**Fig 6:**
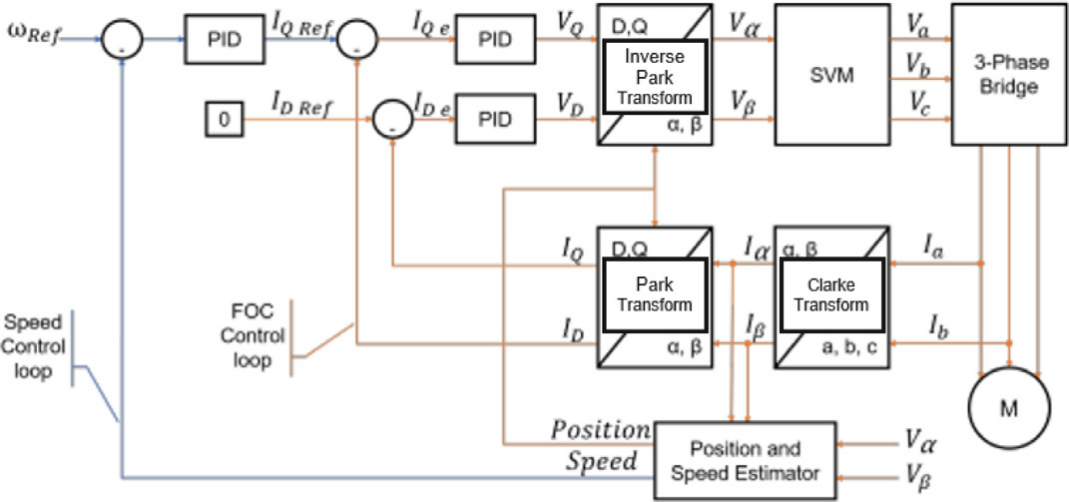
Field Oriented Control Block-scheme representation.

The FOC scheme is shown in Fig. 6, where the acquired signals (defined in Table 2) are labeled for an easier understanding of the process. Some blocks such as the Clarke, Park, and Inverse Clarke transform are also included within the motor control block set. The model implementation in Simulink is based on the sensorless FOC scheme developed by Mathworks^3^, and the controller gains for the PID where obtained using the Motor Control Workbench provided by ST [6].

**Table 2:** Example of 25_step.mat file

SpeedRef	SpeedMeas	Id_ref	Iq_ref	Id_meas	Iq_meas	Vd	Vq
0.03	0.0	0.0	0.00000	0.000488	0.000846	0.15	0.0
0.03	0.0	0.0	0.00825	0.000494	0.000842	0.15	0.0
0.03	0.0	0.0	0.00825	0.010257	0.000131	0.15	0.0
0.03	0.0	0.0	0.00825	0.030278	-0.000105	0.15	0.0
0.03	0.0	0.0	0.00825	0.049320	-0.000606	0.15	0.0

**Table 3:** Motor parameters gathered from the Motor Profiler tool.

Parameter	Abbreviation	Value	Measure unit
Pole Pairs	p	7	-
Max. Speed	*w*_max_	15000	Rpm
Nominal Current	I_n_	1.20	Apk
Nominal DC voltage	V_DC_	12.0	V
Stator Resistance	Rs	0.11	Ohm
Stator Inductance	Ls	0.018	mH
Back-Emf Constant	B-Emf	0.4	Vms/krpm

**Remark:** This FOC scheme requires that that the motor starts in open-loop control, which is achieved when the desired speed is under the 10% of the maximum motor speed. This is considered in every test case, where the first simulation second of every data array corresponds to open-loop control. This data points may be deleted before incurring into ML applications to avoid overfitting with the same data values in every test case instead of considering more valuable data.

### 3.3 Test Cases Generation

In this section, the input signals for the Simulink environment are defined. The test cases are variations of target speed when a step, ramp and random signals are applied to the system; Then, also specific signals are manually generated to verify the controller behavior to nonlinear dynamics.

#### 3.3.1 Step Input

**Fig 7:**
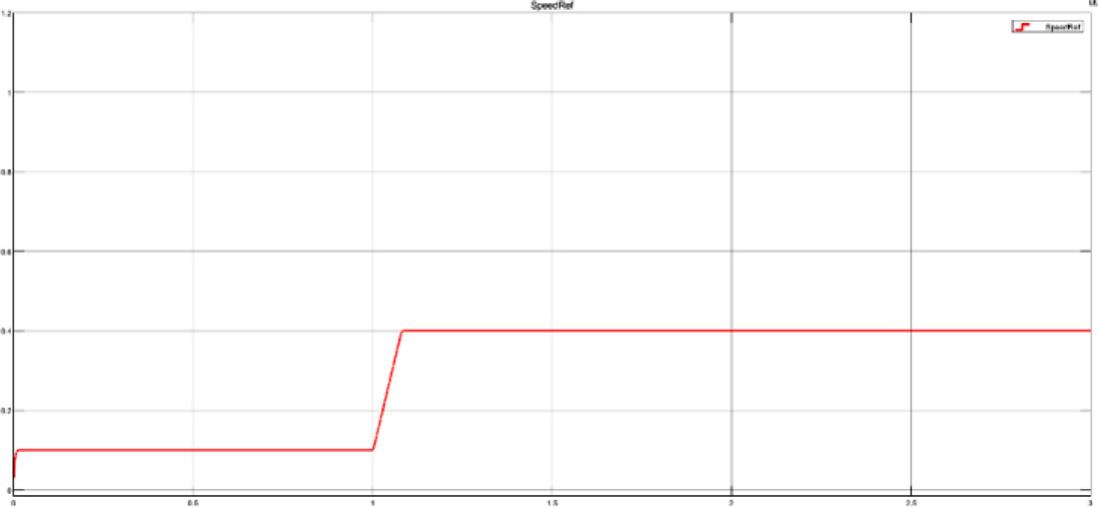
Step input for a 40% speed value.

For the first branch, a step block is used as an input to the FOC scheme where the starting point is the open loop speed (10% of maximum motor speed), and then after one simulation second has elapsed, the final value of the step is the desired motor speed. As an example, the target speed from test case 40_step.mat (Fig. 7) is set up with the following values:

- Initial value: Open loop speed (1500 rpm).
- Step time: 1 second.
- Final value: 40% of the maximum speed value (6000rpm).

The five test cases present in the dataset are related to five different speed targets corresponding to 20, 40, 60, 80, and 100% of the maximum motor speed, which is 15000 rpm as mentioned in previous sections.

#### 3.3.2 Ramp Input

The second branch uses a ramp block for the input signal, the considerations of an open-loop start for the motor still hold. In every test case, the block is parametrized as follows:

- Initial output: Open loop speed (1500 rpm)
- Start time: 1 second (Open loop start of the motor)

**Fig 8:**
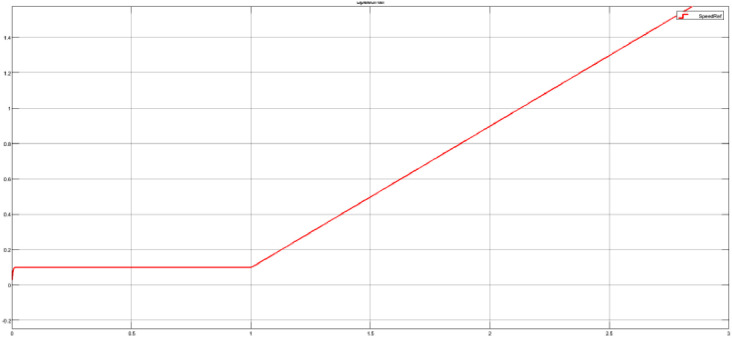
Ramp input with an 80% slope value.

To variate the testing, the slope value in each test case is set to a percentage of the maximum motor speed. An example slope value for the test case 80_ramp.mat is presented below, and the overall target speed signal is shown in Fig. 8.

Case: 80_ramp

- Slope: 80% (Speed increases 12000rpm per second)

#### 3.3.3 Random Signal Input

The third branch uses a random source block for the input signal, this block sets a random mean value in a given range [1500 – 15000 rpm], the signal is generated on this mean value. To guarantee that the motor starts in open-loop control, a step input like the one presented in the test case step_10 is applied with the following values:

- Initial value: Open loop speed (1500 rpm).
- Step time: 1 second (Open loop start of the motor).
- Final value: 0 rpm.

**Fig 9:**
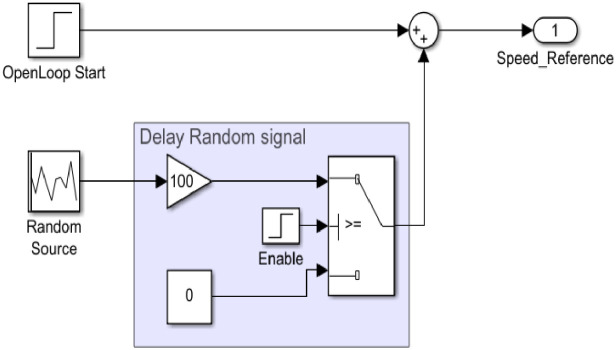
Random signal generator input with delay – Simulink block scheme.

**Fig 10:**
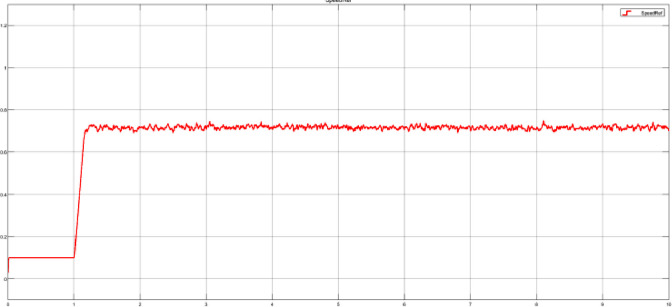
Random signal generated around a mean value of a 70% maximum motor speed.

The random signal needs to be delayed 1 second while the motor is starting in open loop, the developed input block scheme is presented in Fig. 9. Four random signals are included in the dataset, each of which is generated around a different mean value (20%, 40%, 70%, and 100%) of the maximum motor speed. The goal is to verify if the system is able to react at fast speed variations, and also, verify how the controller reacts when having speed variations around low speeds (e.g. 20% mean value) compared to high speed variations (e.g. 100% mean value). As an example, the target speed from test case 70_random.mat is shown in Fig. 10.

#### 3.3.4 Graphically Designed Signal Input

**Fig 11:**
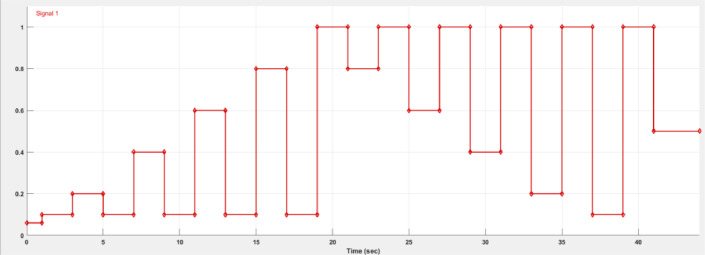
Manually generated signal from the signal builder block.

For the fourth and final branch, the input signal is graphically designed using the “signal builder” block, where the user can graphically generate the desired input signal and adjust its duration. In this test cases the signals were defined by designing high magnitude speed changes, and in some cases, fast speed transitions to bring the system out of control. An example of a generated signal is shown in Fig. 11, during the first second, the motor is under 10% of the maximum speed to start in open loop. **The signal never falls under the 10% speed limit to keep the closed-loop control.**

## Ethics Statement

The authors declare that the data presented in this article did not involve any use of human subjects, animal experiments nor data collected from social media platforms.

## CRediT authorship contribution statement

**Juan Camilo Nustes:** Conceptualization, Methodology, Software, Formal analysis, Investigation, Writing – original draft, Writing – review & editing. **Danilo Pietro Pau:** Conceptualization, Validation, Writing – original draft, Writing – review & editing, Supervision, Project administration. **Giambattista Gruosso:** Conceptualization, Validation, Writing – original draft, Writing – review & editing, Supervision, Project administration.

## Declaration of Competing Interest

The authors declare that they have no known competing financial interests or personal relationships which have, or could be perceived to have, influenced the work reported in this article.

## Data Availability

Motor_control_dataset (Original data) (Mendeley Data).

## Footnotes

2 https://wiki.st.com/stm32mcu/wiki/STM32MotorControl:STM32_MC_Motor_Profiler.

3 https://ww2.mathworks.cn/help/mcb/gs/sensorless-foc-pmsm-smo-fo.html
